# The Relationships between Physical Activity and Life Satisfaction and Happiness among Young, Middle-Aged, and Older Adults

**DOI:** 10.3390/ijerph17134817

**Published:** 2020-07-04

**Authors:** Hsin-Yu An, Wei Chen, Cheng-Wei Wang, Hui-Fei Yang, Wan-Ting Huang, Sheng-Yu Fan

**Affiliations:** 1 Tribal Health Center, Ditmanson Medical Foundation Chia-Yi Christian Hospital, Chia-Yi 600, Taiwan; cych07065@gmail.com; 2 Department of Community Health, Ditmanson Medical Foundation Chia-Yi Christian Hospital, Chia-Yi 600, Taiwan; 07125@cych.org.tw (W.C.); 05791@cych.org.tw (H.-F.Y.); 3 Health Business Development Center, Ditmanson Medical Foundation Chia-Yi Christian Hospital, Chia-Yi 600, Taiwan; cych13649@gmail.com; 4 Clinical Medicine Research Center, Ditmanson Medical Foundation Chia-Yi Christian Hospital, Chia-Yi 600, Taiwan; cych13198@gmail.com; 5 Institute of Gerontology, College of Medicine, National Cheng Kung University, Tainan 701, Taiwan

**Keywords:** exercise, happiness, life satisfaction, life span, physical activity subjective well-being

## Abstract

Physical activity has benefits on physical and psychological health. The aims of this study were to investigate (1) the relationships between physical activity and life satisfaction and happiness in young, middle-aged, and older adults while controlling for demographic characteristics, and (2) the relationships between age and life satisfaction and happiness for different physical activity levels. A total of 2345 healthy adults were recruited. Demographic characteristic, physical activity, life satisfaction, and happiness were collected. Participants were divided into young, middle-aged, and older adult groups based on age, and physical activity was categorized as high, moderate, and low. After controlling for demographic characteristics, participants with high and moderate activity levels had significantly higher life satisfaction and happiness than those with a low activity level across the total population and the three age groups. Age squared was a significant predictor of a positive curvilinear between age and life satisfaction and happiness. Physical activity was significantly related to life satisfaction and happiness in young, middle-aged, and older adults. In addition, life satisfaction and happiness increased with increasing age. The results support the promotion of physical activity.

## 1. Introduction

Subjective well-being, which reflects a good life, is an important issue in social health. Subjective well-being includes two components: cognitive and affective components [1,2]. The cognitive component refers to life satisfaction, and it is a judgment process in which people assess their quality of life based on a personal unique set of criteria [3,4]. The affective component refers to happiness, and it is an emotional appraisal of the degree of intensity and the content of positive personal experiences of the happy moments in a person’s life [4,5].

Physical activity provides people with physical health benefits, for example, improved functional capacity, decreased risks of diseases, improved body composition, and weight loss [6,7]. Previous reviews have also revealed the psychological benefits of physical activity, including improved mood and decreased depression and anxiety [7,8]. In addition, physical activity has broad effects on quality of life [6].

There are positive relationships between physical activity and life satisfaction [9] and physical activity and happiness [10]. A large survey in 24 countries showed that 18–30-year-old young adults with moderate or high physical activity had higher life satisfaction and happiness, and better perceived health [11]. The positive relationship between physical activity and life satisfaction was also founded in older adults [12].

However, there are inconsistent findings in different physical activity intensity levels and subjective well-being. A previous study showed that moderate-to-vigorous intensity physical activity was related to a higher quality of life [13]. In contrast, another study showed that healthy adults gained the highest subjective well-being from low intensity physical activity [14]. The frequency of low and moderate intensity physical activity was related to subjective well-being, but that of high intensity physical activity was not [15]. A prospective cohort study showed that the number of days people participated in moderate intensity physical activity was positively related to subjective well-being; however, the time spent on high intensity physical activity was negatively related to subjective well-being [16]. It is unknown whether the total amount of physical activity or a specific physical activity has positive effects on life satisfaction and happiness.

There are inconsistent findings on trends in life satisfaction and happiness across the lifespan. A review supported that life satisfaction was stable across different stages of the lifespan [1], whereas another study showed an inverted U relationship between age and life satisfaction, with a lower life satisfaction during emerging and older adulthood and a higher life satisfaction during young and middle-aged adulthood [9]. The study also showed that adults who performed more physical activity had a higher life satisfaction, and the effect of physical activity was stronger in older adults than in young adults [9].

There was a similar inconsistency in happiness and age; for example, happiness decreased with increasing age [17,18], and older adults were less happy [19]. There was a U-shaped relationship with happiness decreasing from age 45 to 54 and increasing from age 55 to 64 [20]. The effects of different amounts of physical activity on life satisfaction and happiness remain unknown.

Demographic characteristics are significantly related to life satisfaction and happiness. Female gender [17,21], a marital status of married or partnered [17,19,20,21], a higher education level [18,22], and a higher income level [18,19] tend to be associated with higher subjective well-being or happiness. In this study, demographic characteristics were considered covariates.

Previous studies have shown the potential benefits of physical activity on objective health indices, but few studies have focused on subjective indices, including life satisfaction and happiness. In addition, few studies have compared the effects across the lifespan. Therefore, the first purpose of this study was to investigate the relationships between physical activity and life satisfaction and happiness in young, middle-aged, and older adults while controlling for demographic variables. This study aimed to test whether the time spent performing physical activity of different levels (i.e., vigorous, moderate, and low) or the total amount of physical activity could significantly predict life satisfaction and happiness. The second purpose was to investigate the relationships between age and life satisfaction and happiness for different physical activity levels. The study aimed to test whether the relationships between age and life satisfaction and happiness were linear or curvilinear for the three different physical activity levels.

## 2. Methods

### 2.1. Participants

This study was a cross-sectional study, which recruited a large sample size to explore the effects of physical activity on life satisfaction and happiness cross lifespan. The inclusion criteria for the participants were adults aged >18 years who could communicate and complete the questions. The potential participants were recruited from a citizen physical fitness examination held by the Sports Administration of the Ministry of Education in Chia-Yi, a city in Southern Taiwan. Data were collected in 12 examination stations from January 2016 to November 2017. Adults who had serious diseases and communication problems were excluded. Ethical approval was obtained from the institutional review board of a teaching hospital (IRB number: 2020024).

### 2.2. Data Collection

#### 2.2.1. Demographic Characteristics

Demographic characteristics included age, gender, educational level, marital status, monthly income, and whether or not participants lived alone. Monthly income was rated on a 5-point scale (1 = less than 20,000 New Taiwan dollars (TWD); 2 = TWD 20,001–40,000; 3 = TWD 40,001–60,000; 4 = TWD 60,001–80,000; and 5 = more than TWD 80,000).

#### 2.2.2. Life Satisfaction and Happiness

Life satisfaction was evaluated with a single question whose response was rated on an 11-point scale from 0 to 10: “In general, how satisfied are you with your life?” This single item measure was significantly correlated with the full scale of the Satisfaction with Life Scale [23]. Happiness was also evaluated with a single question rated on an 11-point scale: “Do you feel happy in general?” The item had good convergent and divergent validity [24]. Higher scores meant better life satisfaction and happiness.

#### 2.2.3. Physical Activity

The International Physical Activity Questionnaire (IPAQ) short form was used to evaluate the participants’ physical activity levels [25]. Participants were asked to report the days and times they performed physical activity during the last seven days at three intensities: vigorous, moderate, and low. Examples of vigorous intensity activities include heavy lifting or digging, and moderate intensity activities include carrying light loads or bicycling at a regular pace. Low intensity activities included walking. The total weekly physical activity was estimated weighting the time spent performing each activity intensity with its metabolic equivalent (MET) energy expenditure. The METs of vigorous, moderate, and low intensity activities were 8.0, 4.0, and 3.3 METs, respectively. Based on the total METs, the participants were divided into low-active (0 ≤ METs < 600), moderate-active (600 ≤ METs < 3000), and high-active (METS ≥ 3000) groups [25]. The IPAQ has been translated into Taiwanese with a good reliability and validity [26]. The participants rated life satisfaction and happiness first, and then completed the IPAQ.

### 2.3. Statistical Analysis

The participants were divided into three age groups: young (age 18–44), middle-aged (age 45–64), and older (age 65 and above) adults. Descriptive statistics were used to present the demographic characteristics of the total population and participants in the three age groups.

Multiple hierarchy regression was used with life satisfaction and happiness as dependent variables, and demographic characteristics and physical activity variables were entered as predictors. Categorical variables, including gender, educational level, and marital status, were recoded as dummy variables. In the young and middle-aged groups, the proportions of participants with an elementary school or a junior high school education level were too small and were combined, and the same was done for an undergraduate and postgraduate education level in the older adult group.

Demographic characteristics were entered in the first model, and the time of vigorous, moderate, and low intensity activities (continuous variables) and the physical activity groups (categorical variables) were entered in the second model. Regarding the relationships between age and life satisfaction and happiness, age, and age squared (age^2^), these were entered together [18,21]. While age was significant, the relationship was linear, and life satisfaction and happiness can be predicted by that age multiplied by the regression coefficient. While age squared was significant, life satisfaction and happiness can be predicted by that age^2^ multiplied by the regression coefficient. Age changed 1 unit and life satisfaction and happiness changed more; therefore, the relationship was a curvilinear relationship. A variance inflation factor > 10 was used to examine the degree of multicollinearity. In addition, a line of fit was used to present the relationships between age and life satisfaction and happiness in participants with the three physical activity levels.

A two-tailed *p*-value < 0.05 indicated a statistically significant difference. SPSS software for Windows, version 17 (IBM Corporation, Somers, NY, USA) was used for statistical analysis.

## 3. Results

### 3.1. Demographic Characteristics

A total of 2345 healthy adults were recruited. The mean age was 51.06 years (SD = 17.01), and 67.85% were female. Approximately 50% had an undergraduate degree; 66.78% were married; and 13.52% lived alone. The percentages of low-active, moderate-active, and high-active participants were 42.77%, 21.88%, and 35.35%, respectively. In the young adult group, the mean age was 34.60 years (SD = 6.17), and most were female (67.55%), had an undergraduate degree (68.68%), were married (50.26%), lived with family (87.72%), and had a low activity level (50.87%). In the middle-aged adult group, the mean age was 53.15 years (SD = 5.34), and most were female (74.90%), had a postgraduate degree (43.59%), were married (78.76%), lived with family (91.17%), and had a low activity level (45.52%). In the older adult group, the mean age was 73.71 years (SD = 5.94), and most were female (60.34%), had an elementary school education, were married (78.38%), lived with family (79.32%), and had a high activity level (44.01%) (Table 1).

### 3.2. Life Satisfaction

Compared with the low-active group, the high-active (Beta = 0.19, *p* < 0.001) and moderate-active groups (Beta = 0.12, *p* < 0.001) had significantly higher life satisfaction. Age squared (Beta = 0.34, *p* = 0.026) rather than age was significant. The other significant predictors were having an undergraduate (Beta = 0.14, *p* = 0.001) or postgraduate degree (Beta = 0.09, *p* = 0.005) and being married (Beta = 0.13, *p* < 0.001).

In young adults, the significant predictors were a high (Beta = 0.23, *p* < 0.001) and moderate activity level (Beta = 0.11, *p* = 0.001) and being married (Beta = 0.13, *p* = 0.001). In middle-aged adults, the significant predictors were a high (Beta = 0.17, *p* = 0.001) and moderate activity level (Beta = 0.11, *p* = 0.007), having an undergraduate (Beta = 0.20, *p* = 0.001) and postgraduate degree (Beta = 0.12, *p* = 0.016), being married (Beta = 0.17, *p* < 0.001), and monthly income (Beta = 0.13, *p* = 0.002). In older adults, the significant predictors were a high (Beta = 0.18, *p* = 0.004) and moderate activity level (Beta = 0.14, *p* = 0.007), having an undergraduate or postgraduate degree (Beta = 0.12, *p* = 0.016), and living alone (Beta = −0.10, *p* = 0.025) (Table 2).

### 3.3. Happiness

Compared with the low-active group, the high-active (Beta = 0.19, *p* < 0.001) and moderate-active groups (Beta = 0.09, *p* < 0.001) were happier. Age squared (Beta = 0.26, *p* = 0.049) rather than age was significant. The other significant predictors were having an undergraduate degree (Beta = 0.10, *p* = 0.016) and being married (Beta = 0.09, *p* < 0.001).

In young adults, the significant predictors were a high (Beta = 0.16, *p* = 0.001) and moderate activity level (Beta = 0.08, *p* = 0.022) and being married (Beta = 0.10, *p* = 0.017). In middle-aged adults, the significant predictors were a high (Beta = 0.19, *p* < 0.001) and moderate activity level (Beta = 0.07, *p* = 0.040), having an undergraduate (Beta = 0.21, *p* = 0.001) or postgraduate degree (Beta = 0.15, *p* = 0.003), being married (Beta = 0.13, *p* = 0.001), and monthly income (Beta = 0.10, *p* = 0.014). In older adults, the significant predictors were a high (Beta = 0.21, *p* = 0.001) and moderate activity level (Beta = 0.11, *p* = 0.024) and having an undergraduate or postgraduate degree (Beta = 0.10, *p* = 0.045) (Table 3).

**Figure 1:**
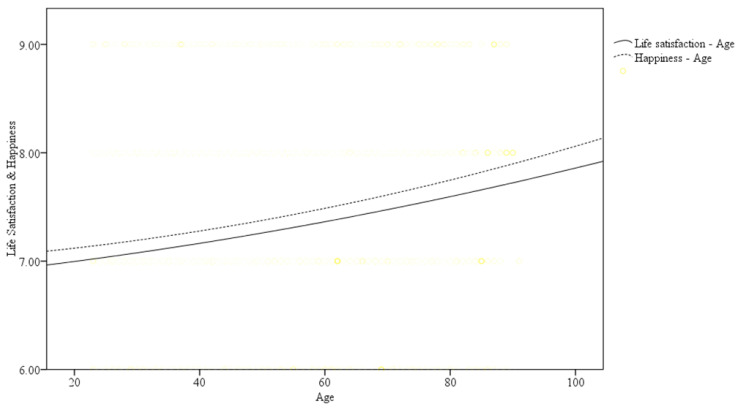
Estimated age patterns for life satisfaction in adults based on the three physical activity levels.

Because age squared was significant in life satisfaction and happiness, curvilinear lines of fit were used to present the relationships between age and life satisfaction and happiness in the total participants (see Figure 1). Participants in the high-active and moderate-active groups had better life satisfaction and were happier across the lifespan.

## 4. Discussion

After controlling for demographic characteristics, physical activity was significantly related to life satisfaction and happiness across the three age groups. Participants with a higher physical activity level tended to have higher life satisfaction and happiness. The positive relationships between age and life satisfaction and happiness were curvilinear. In addition, education was also a significant variable for middle-aged and older adults; marriage was a significant variable for young and middle-aged adults; and monthly income was only a significant variable for middle-aged adults.

Exercise or physical activity makes people feel good or happy [8,10], and the effects in this study occurred across the lifespan. The total amount of physical activity was more important than the specific type of physical activity, which did not support the findings of a previous study [16]. Overall lifestyle was more important than a single physical activity. Therefore, people should modify their lifestyle.

There are two potential mechanisms for the effects of physical activity on subjective well-being. The first is through the improvement of physical health, including cardiovascular status, strength, and functional capacity. The second is through changes in psychological variables, such as decreased anxiety and depression and increased self-efficacy, view of oneself, and mental health [6,9,27,28]. In addition, people with mental health issues could derive higher life satisfaction from exercise.

There was a bidirectional association between physical activity and life satisfaction/happiness [29]. Physical activity was one of important healthy lifestyles, which could improve physical and mental health, and then increase life satisfaction and happiness. On the other hand, happiness might be a protective factor to health [30], and people with higher life satisfaction/happiness might participate in more physical activity [31].

Similar to the findings of previous studies [9,18,32], the relationships between age and life satisfaction and happiness were U-shaped. Life satisfaction and happiness increased with increasing age, but when getting older, life satisfaction and happiness increased more. Aging is associated with more positive emotions from early adulthood to old age, and emotional experiences grow more stable [33]. They might not have stress from life events and economic problems. However, the participants in this study had good mobility and health. The results did not consider the effects of health status or disease.

Similar to the population in previous studies [18,22], participants with higher educational degrees tended to have higher life satisfaction and happiness. People with higher education had more health knowledge or a better ability to deal with life problems. The results of this study demonstrated the advantage of education in middle-aged and older adults, rather than in young adults. Due to social development, more than 80% of young adults in this study had undergraduate or postgraduate degrees. However, not all studies support this point. Some studies revealed that education was not significantly related to subjective well-being [17,21], and education may have indirectly affected subjective well-being through health [20].

Participants who were married had higher satisfaction and happiness [17,18,20,22]. People who are married receive a variety of physical, emotional, social, and economic benefits. Marriage is a normative achievement that is positively viewed by oneself and others, which leads to greater happiness and life satisfaction [34]. However, the positive effects of marriage only occurred in young and middle-aged adults. Older adults may be widowed or divorced and may have experienced the long-term negative effects of marriage, such as strain or conflicts between spouses. In addition, older adults who lived alone had lower satisfaction than those who lived with family or friends. Emotional and practical support from adult children may play an important role in older adults’ life satisfaction [35].

Personal income reflects one’s financial status, which was related to life satisfaction and happiness. People with a low income or who are unemployed have lower life satisfaction [36]. Monthly income was particularly important for middle-age adults because these individuals play an important role in earning money for their family in this life stage. One of the major life tasks in middle age is earning money. Therefore, middle-aged adults may experience economic stress.

The strength of this study was its large sample size from a citizen health examination. The results support the promotion of physical activity, and the amount of physical activity was more important than the type of physical activity. However, the participants recruited from the health examination had good mobility, and those who had mobile problems or serious diseases were not included. Some covariate variables, including physical and mental health status, diseases, and medication history, were not controlled. In addition, this study was a cross-sectional study that compared the differences between age groups. Personal life experiences, aging effects, or cohort effects may influence the long-term trend of life satisfaction and happiness. The causal effects of physical activity on life satisfaction and happiness could not be demonstrated. In future studies, a longitudinal study design with comprehensive measures of life satisfaction, happiness, and covariate variables can be used.

## 5. Conclusions

Higher physical activity was significantly related to better life satisfaction and happiness in young, middle-aged, and older adults. In addition, there was a positive curvilinear between age and life satisfaction and happiness, in which life satisfaction and happiness increased with increasing age. The results support the promotion of physical activity.

## Figures and Tables

**Table 1:** Demographic characteristics of adults in the three age groups.

	Total (*n* = 2345)	Young Adults (*n* = 977)	Middle-Aged Adults (*n* = 725)	Older Adults (*n* = 643)
Age, mean (SD)	51.06 (17.01)	34.60 (6.17)	53.15 (5.34)	73.71 (5.94)
Gender				
Male	754 (32.15)	317 (32.45)	182 (25.10)	255 (39.66)
Female	1591 (67.85)	660 (67.55)	543 (74.90)	388 (60.34)
Education level				
Elementary school	337 (14.37)	4 (0.41)	46 (6.34)	287 (44.63)
Junior high school	164 (6.99)	22 (2.25)	65 (8.97)	77 (11.98)
Senior high school	496 (21.15)	140 (14.33)	228 (31.45)	128 (19.91)
Undergraduate	1127 (48.06)	671 (68.68)	316 (43.59)	140 (21.77)
Postgraduate	221 (9.42)	140 (14.33)	70 (9.66)	11 (1.71)
Marital status				
Single	589 (25.12)	460 (47.08)	107 (14.76)	22 (3.42)
Married	1566 (66.78)	491 (50.26)	571 (78.76)	504 (78.38)
Other	190 (8.10)	26 (2.66)	47 (6.48)	117 (18.20)
Living alone				
No	2028 (86.48)	857 (87.72)	661 (91.17)	510 (79.32)
Yes	317 (13.52)	120 (12.28)	64 (8.83)	133 (20.62)
Physical activity level				
Low-active	1003 (42.77)	497 (50.87)	330 (45.52)	176 (27.37)
Moderate-active	513 (21.88)	175 (17.91)	154 (21.24)	184 (28.62)
High-active	829 (35.35)	305 (31.22)	241 (33.24)	283 (44.01)

SD, standard deviation.

**Table 2:** Summary of regression analyses: demographic characteristics, physical activity, and life satisfaction.

	Total (*n* = 2345)		Young Adults (*n* = 977)		Middle-Aged Adults (*n* = 725)		Older Adults (*n* = 643)	
	Model 1	Model 2	Model 1	Model 2	Model 1	Model 2	Model 1	Model 2
Age	−0.23	−0.22	−0.54	−0.38	1.00	1.06	0.14	0.16
Age square (Age ^2^)	0.39 *	0.34 *	0.51	0.35	−0.94	−1.01	−0.17	−0.17
Gender								
Male	REF	REF	REF	REF	REF	REF	REF	REF
Female	0.02	0.03	−0.02	−0.01	0.04	0.06	0.03	0.04
Education level								
Elementary school	REF	REF	REF	REF	REF	REF	REF	REF
Junior high school	0.03	0.03	-- ^a^	-- ^a^	-- ^a^	-- ^a^	0.08	0.07
Senior high school	0.02	0.02	−0.03	−0.05	0.01	0.02	0.07	0.05
Undergraduate	0.14 **	0.14 **	0.02	−0.00	0.19 **	0.20 **	0.14 **	0.12 *
Postgraduate	0.10 **	0.09 **	0.04	0.01	0.12 *	0.12 *	-- ^b^	-- ^b^
Marital status								
Single or Other	REF	REF	REF	REF	REF	REF	REF	REF
Married	0.12 ***	0.13 ***	0.12 **	0.13 **	0.17 ***	0.17 ***	0.05	0.06
Living alone								
No	REF	REF	REF	REF	REF	REF	REF	REF
Yes	−0.03	−0.03	−0.02	−0.01	0.02	0.02	−0.11 *	−0.10 *
Monthly income	0.05	0.05	0.04	0.04	0.13 *	0.13 **	−0.08	−0.09
Time of vigorous activity		0.02		−0.04		0.03		0.06
Time of moderate activity		−0.04		−0.06		−0.01		−0.04
Time of walking		−0.02		−0.04		−0.01		−0.02
Physical activity level								
Low-active		REF		REF		REF		REF
Moderate-active		0.12 ***		0.11 **		0.11 **		0.14 **
High-active		0.19 ***		0.23 ***		0.17 **		0.18 **

REF, reference. * *p* < 0.05, ** *p* < 0.01, *** *p* < 0.001. ^a^: The reference education group for the young and middle-aged adult groups was elementary school and junior high school. ^b^: The highest education level in the older adults group was the combination of undergraduate and postgraduate levels.

**Table 3:** Summary of regression analyses: demographic characteristics, physical activity, and happiness.

	Total (*n* = 2345)		Young Adults (*n* = 977)		Middle-Aged Adults (*n* = 725)		Older Adults (*n* = 643)	
	Model 1	Model 2	Model 1	Model 2	Model 1	Model 2	Model 1	Model 2
Age	−0.16	−0.16	−0.74	−0.62	0.77	1.01	1.30	1.28
Age square (Age ^2^)	0.30 *	0.26 *	0.69	0.57	−0.65	−0.90	−1.36	−1.34
Gender								
Male	REF	REF	REF	REF	REF	REF	REF	REF
Female	0.02	0.04	0.00	0.01	0.04	0.06	0.03	0.04
Education level								
Elementary school	REF	REF	REF	REF	REF	REF	REF	REF
Junior high school	−0.00	−0.00	-- ^a^	-- ^a^	-- ^a^	-- ^a^	0.05	0.05
Senior high school	0.02	0.02	−0.06	−0.07	0.08	0.09	0.05	0.04
Undergraduate	0.10 *	0.10 **	−0.03	−0.04	0.21 **	0.21 **	0.11 *	0.10 *
Postgraduate	0.05	0.05	−0.05	−0.07	0.14 **	0.15 **	-- ^b^	-- ^b^
Marital status								
Single or Other	REF	REF	REF	REF	REF	REF	REF	REF
Married	0.08 **	0.09 ***	0.09 *	0.10 *	0.13 **	0.13 **	0.02	0.02
Living alone								
No	REF	REF	REF	REF	REF	REF	REF	REF
Yes	−0.04	−0.03	−0.05	−0.05	0.02	0.02	−0.09	−0.08
Monthly income	0.02	0.02	0.00	−0.00	0.11 **	0.10 *	−0.08	−0.08
Time of vigorous activity		−0.01		−0.06		0.01		0.01
Time of moderate activity		−0.02		−0.02		0.01		−0.06
Time of walking		−0.03		−0.02		−0.03		0.00
Physical activity level								
Low-active		REF		REF		REF		REF
Moderate-active		0.09 ***		0.08 *		0.07 *		0.11 *
High-active		0.19 ***		0.16 **		0.19 ***		0.21 **

REF, reference. * *p* < 0.05, ** *p* < 0.01, *** *p* < 0.001. ^a^: The reference education group for the young and middle-aged adult groups was elementary school and junior high school. ^b^: The highest education level in the older adults group was the combination of undergraduate and postgraduate levels.
